# Symptom-Based Identification of G-4 Chili Leaf Diseases Based on Rotation Invariant

**DOI:** 10.3389/frobt.2021.650134

**Published:** 2021-05-28

**Authors:** Sufola Das Chagas Silva Araujo, V. S. Malemath, K. Meenakshi Sundaram

**Affiliations:** ^1^ Laboratory KLE, Institute KLE Dr. M. S. S. Sheshgiri College of Engineering and Technology, Department of Computer Science Engineering, Organization: Visvesvaraya Technological University, Udyambag, India; ^2^ Institute KLE Dr. M. S. S. Sheshgiri College of Engineering. and Technology, Department of Computer Science Engineering, Organization: Visvesvaraya Technological University, Udyambag, India; ^3^ Institute Botho University, Department of Computer Science Engineering, Organization: Botho University, Gaborone, Botswana

**Keywords:** Guntur-4, infection, leaf, symtoms, moments, corelation

## Abstract

Instinctive detection of infections by carefully inspecting the signs on the plant leaves is an easier and economic way to diagnose different plant leaf diseases. This defines a way in which symptoms of diseased plants are detected utilizing the concept of feature learning (Sulistyo et al., 2020). The physical method of detecting and analyzing diseases takes a lot of time and has chances of making many errors (Sulistyo et al., 2020). So a method has been developed to identify the symptoms by just acquiring the chili plant leaf image. The methodology used involves image database, extracting the region of interest, training and testing images, symptoms/features extraction of the plant image using moments, building of the symptom vector feature dataset, and finding the correlation and similarity between different symptoms of the plant (Sulistyo et al., 2020). This will detect different diseases of the plant.

## Introduction

Recognition of infection in plants is significant. To notice infection at early stages, we require different infection-revealing techniques (Singh and Misra, 2017). At present, infection and disease detection in plants is done via simple naked eye observation by experts or by taking samples from those plants and observing them carefully under highly sophisticated microscopes to determine exactly which virus is causing the disease. For this, a large team of professionals and endless observing of chili plant growth are a must, which leads to high overheads as the size of farms increases (Singh and Misra, 2017). In many countries, agriculturalists do not have proper amenities or even awareness of how to contact the authorities (Singh and Misra, 2017). Due to this, consulting specialists are expensive. The scientists are detecting these diseases manually, which is more prone to human errors and also time consuming.

So, a better way is to use a system that can detect whether a plant is suffering from a disease or not. In such conditions, an automatic system has been shown to be more gainful in checking large fields of crops (Singh and Misra, 2017).

Manual revealing is more troublesome because human eyes have to detect the symptoms and the disease based on shape and color. The system proposed to detect the different diseases automatically. This will give less error prone results within a less period of time. The scientists have to detect the infected leaves of the plant manually. This can be time-consuming and can have human errors. Manual detection is more troublesome because human eyes have to detect the region of interest based on the shape and color of the infected area (Sulistyo et al., 2020). The proposed method will detect the different symptoms and detect the different diseases. This will give less error prone results within a shorter amount of time.

When diseases attack the plant, the overall yield reduces, and sometimes, it kills the plant (Sufola et al., 2019). In the last few years, as per the Indian Council of Agricultural Research (I.C.A.R), Goa, growth of G-4 (Guntur-4) variety of chilies in Goa has decreased radically due to some type of disease attacking the plants (Sufola et al., 2019). Most chili plants start off with poor flowering of the plants and sometimes, no flowering at all (Sufola et al., 2019). In erratic cases, when the plant flowers, the produce is noticeably poor (Sufola et al., 2019). So, this model is devised to identify the occurrence of infection in the chili plant by inspecting the symptoms on the foliage (Sufola et al., 2019).

## System Proposed

The system developed is an automated system, where it detects the different symptoms in the diseased leaf of the plant. It enables the user to capture an image, detect, and recognize whether the crop is infected or not. We create an object detection model to detect diseases in leaves of chili plants.

We train a model to classify leaves as infected or not and detect the symptoms to detect the type of infection in the chili plant.

**FIGURE 1:**
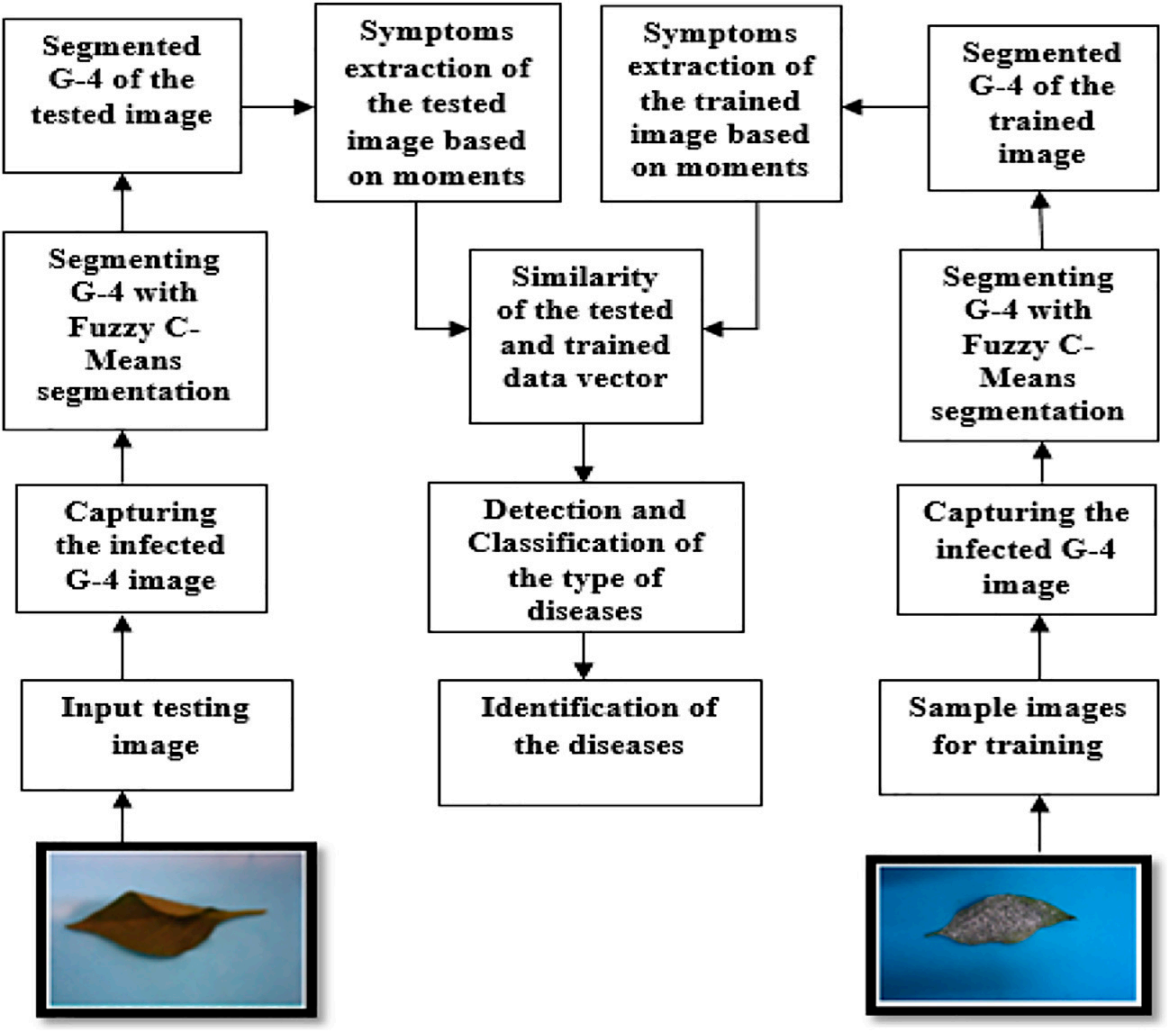
System flowchart to detect disease in chili plants.

This process has different steps. These steps are shown in the following diagram (Figure1). The input is an image of a chili leaf which may be infected or not infected. The output of the system will identify the symptoms and detect the disease. This model extracts symptoms using moments for object finding, to categorize crops as infected with different diseases (Sufola et al., 2019).

## Methodology

The methodology of the proposed system to detect different symptoms to check for plant infection is given below (Hemanth et al., 2019; Ramya et al., 2015).

### Image Collection

Images were attained from the fields of G-4 (Guntur-4) variety chili plants, which in this case were from the I.C.A.R (Indian Council of Agricultural Research) (Hemanth et al., 2019). Scientists and associate researchers prepared the fields of the variety of plants that gets affected every year to find out the cause of the infection so that the farmers do not suffer a terrible produce. Chili leaf images were captured using a digital camera in a mini photo box of 30 cm * 45 cm * 35 cm. A total of 7,850 digital images of G-4 variety of chili leaves were captured, out of which 1,554 images were of bacterial leaf spot infection, 1,568 of powdery mildew (whitefly), 1,570 of chili leaf curl, 1,577 of *Fusarium* wilt (yellow), and 1,581 of healthy leaves.

The total data set of 7,850 images of chili leaves was divided into 5,000 images used as a training set and 2,850 images used for testing (Sufola et al., 2020). So the proposed method was tested on the data set to distinguish between these five different classes so as to identify the G-4 variety chili leaves to their respective class (Sufola et al., 2020).

### Pre-Processing

The image was resized to 256 × 256 pixel (Dey et al., 2014). Upon resizing, the image was passed through a Gaussian filter to overcome any noise present (Pandian et al., 2019).

**FIGURE 2:**
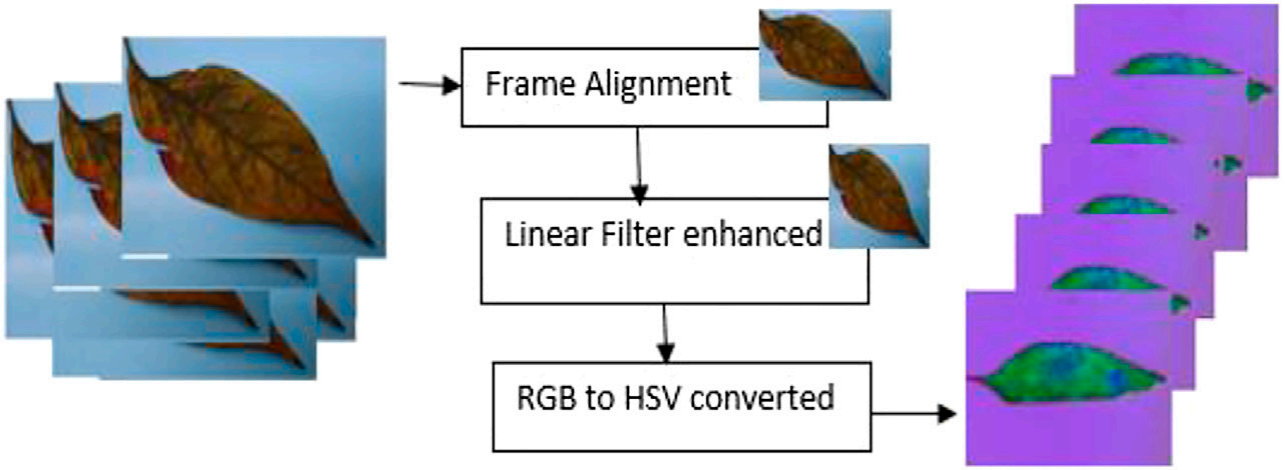
G-4 chili leaf input image, resized, filtered, and HSV.

The RGB image was converted to HSV in order to make the disease identification easier (Pandian et al., 2019). Figure 2 shows the preprocessing cycle for the G-4 chili leaf input image, resized, filtered, and RGB to HSV converted.

### Image Segmentation

The images which were obtained were given as input to a segmentation algorithm (Nandibewoor and Hegadi, 2019). Segmentation of an image is clustering similar property pixels into one cluster (Sufola et al., 2016a; Sufola et al., 2019; Ramya et al., 2015). The segmentation was done so that the image was represented in a more meaningful way so that it became easier to analyze (Sufola et al., 2018). Segmentation was done to separate the wanted part of the image from the background (Nandibewoor and Hegadi, 2019). Segmentation helps to distinguish the region of interest from the background of the image. The segmentation and clustering algorithm used was fuzzy c-means (FCM).

### Fuzzy C-Means Segmentation Algorithm

Step 1: Number of clusters, the fuzzy parameter (a constant >1), and the stopping condition are set.
Step 2: Fuzzy partition matrix is initialized.
Step 3: Loop counter is set.
Step 4: Cluster centroids and the objective value J are computed.
Step 5: Membership values in the matrix are computed.
Step 6: When the value of J between iterations is less than the predefined stopping condition, stop, or else, increment k and go to step 4.
Step 7: De-fuzzification and segmentation.

The fuzzy c-means algorithm extracted the region of interest from the chili leaf image, where the object needed is a member of multiple clusters, with degrees of membership changing between 0 and 1 in FCM (Sufola et al., 2016b; Singh & Misra, 2017; Dey et al., 2014).

### Trained Images

It is a data set of images which had different types of images of the infected and not infected leaves of the G-4 (Guntur-4) variety chilies. A total of 5,000 images out of 7,850 images were used as the training data set. These images were used to create the feature vector for training the proposed method.

### Testing Data Images

The remaining 2,850 images from 7,850 were used as the testing data set. These images were from all five classes: bacterial leaf spot, powdery mildew (whitefly), chili leaf curl, *Fusarium* wilt (yellow), and healthy leaves.

Testing was done on the image to check if it was of any of the five types of chili leaves. The feature vector of these images was compared against the standard trained feature vector in the feature vector dataset.

### Feature Extraction

This was a very important phase of this project. Feature extraction includes morphological operations (Pandit et al., 2015). The features are based on shape, color, and size (Sapna and Renuka, 2017). It extracts some important information of the object of interest (Titlee et al., 2017).

The feature vector of the trained image and the feature vector obtained from the test image were compared (Leeson, 2003; Ramya et al., 2015). The features were obtained by calculating the moments of each region. The following are the steps to calculate feature vector:

### Dividing the Image Into Regions

Calculate area of each region.

Calculate the number of x-coordinate pixels and y-coordinate pixels of each region.

Calculate x- and y-centroids of each region.

Calculate x- and y-centroids of entire image.

### Dividing the Image Into Regions

After segmenting the image, the image was divided into regions. This step is needed since it makes it easy to get the region of interest. It also becomes easier to get the area and centroid of the region of interest.

### Calculating Area of Each Region

Here, we count the number of pixels which satisfies some condition.

The formula for it is as follows:

$$area=\sum f\left(x,y\right)\left\{\frac{+1\;\;if\left(g<90\&\&r>5\&\&b>5\right)}{+0\;\;if\left(g<90\&\&r>5\&\&b>5\right)}\right\},$$

where f (x, y) is the measure of the pixel at coordinates x and y (Dhivyaprabha et al., 2018) and x is the height and y is the width of each block made of g (green), r (red), and b (blue) measures of pixels (Mothkur & Poornima, 2018; Persson & Åstrand, 2008).

1) Calculate the number of x-coordinate pixels and y-coordinate pixels

Here, we are summing up all the values of x-coordinate pixels and y-coordinate pixels, which satisfy the above condition.

2) Calculating x-centroid and y-centroid of each region

The center along the *x* and *y* axes of each region is calculated.

The following is the formula to calculate the x-centroid of each region:

$$xcentroid=\left(\frac{sumx}{area}\right),$$

where sumx is the number of pixels in the x-coordinate and area is the area of each region.

The following is the formula to calculate the y-centroid of each region:

$$ycentroid=\left(\frac{sumy}{area}\right),$$

where sumy is the number of pixels in the y-coordinate and area is the area of each region.

3) Calculating the x-centroid and y-centroid of the entire image

Here, it calculates the x-centroid and y-centroid of the entire image region.

4) Calculating the feature vector

Here, we are calculating the feature vector. Feature vector is the vector of moments.

Moments:

Image moment is the average or moment of an image pixel’s intensities or moment function, which usually has some properties related to the image. These properties can be area, centroid, and so on. Moments are suitable in shape learning. Zero to second order moments are applied for shape learning and orientation (Jacobs and Bean, 1963).

The formula to calculate moment is as follows:

$$mpg=\underset{x}{\sum }\underset{y}{\sum }x^{p}y^{q}.$$

Here, *p*, q = 0,1,2. x = (number of x-coordinate pixel)–(xloc) (Gabriel, 2010). y = (number of y-coordinate pixel)–(yloc) (Gabriel, 2010).

#### The zeroth order moment gives the information of the area in the foreground, or it counts the total number of pixels in the region of interest.

M_10_ is the measure of first order moment at *x*-axis (Pradhan and Deepak, 2016; Titlee et al., 2017; Schoeffmann et al., 2006; Adams et al., 2018).

M_01_ is the measure of first order moment at *y*-axis (Pradhan and Deepak, 2016; Titlee et al., 2017; Schoeffmann et al., 2006; Adams et al., 2018).

M_20_ is the measure of second order moment at *x*-axis (Adams et al., 2018; Schoeffmann et al., 2006).

M_02_ is the measure of second order moment at *y*-axis (Adams et al., 2018; Schoeffmann et al., 2006).

#### Feature Vector Data Set

It is the data set which had the name of the infection, the file path, and the feature of that infected image.

#### Similarity of Regions

The symptoms of the images stored as feature vectors in the trained database were compared to the feature vector generated from the image under test to obtain a match. The feature vector of the test image was associated with the feature vector of the different infected images in the trained image feature data set. To relate the two feature vectors, the coefficient of correlation (CoC) of the two feature vectors was calculated.

Coefficient of correlation helps in judging resemblances between two measured vector quantities, while analyzing whether the two quantities are identical or completely different. Pearson’s correlation coefficient is denoted as *r* and is used in shape learning and computer image identification (Kaur et al., 2012; Anilkumar et al., 2020) (Anilkumar et al., 2020).

Steps to calculate the coefficient of correlation are as follows:

Considering two feature vectors u,v.

(1) Finding the average of the two feature vectors u,v.
(2) Calculating the difference vector of u,v.
(3) Calculating unit vector.
(4) Calculating correlation (similarity) of the two unit vectors.
(1) Finding the average of the two feature vectors u,v. We determine the average of the feature vectors by using

$$\bar{u}=\left(\frac{\sum_{1}u_{i}}{n}\right).$$

n = Quantity of values in vector u (Pandian et al., 2019); i = I, 2, 3 …n (Pandian et al., 2019).

$$\bar{v}=\left(\frac{\sum_{i}v_{i}}{n}\right).$$

n = quantity of values in vector v; i = 1, 2, 3 … n (Dhivyaprabha et al., 2018).

(2) Calculating the difference vector of u,v.

Here, we subtract each element of the feature vector from the average of the feature vector.

The formula to find the difference vector for feature vector u is as follows:

$$\left\{u\right\}-\bar{u}.$$

The formula to find the difference vector for feature vector v is as follows:

$$\left\{v\right\}-\bar{v}.$$

Here {u} is symptom vector (Zhou et al., 2015). {v} is symptom vector (Zhou et al., 2015). $\bar{u}$ is the average symptom vector u (Hsiao, 2007). $\bar{v}$ is the average symptom vector v (Hsiao, 2007).

(3) Calculating unit vector.

Here, we calculate the unit vector, which is the difference vector divided by the length.

The formula for calculating the length of the vector u is as follows:

$$\left|u\right|=\sqrt{u_{1}^{2}+u_{2}^{2}+\ldots +u_{n}^{2}}.$$

Here, |u| is the length of the u vector. $u_{1}^{2}+u_{2}^{2}+\ldots +u_{n}^{2}$ is the element of the vector u.

The formula for computing the dimension of the vector v is as follows:

$$\left|v\right|=\sqrt{v_{1}^{2}+v_{2}^{2}+\ldots +v_{n}^{2}}.$$

Here, |v| is the length of the v vector. $v_{1}^{2}+v_{2}^{2}+\ldots +v_{n}^{2}$ is the element of the vector v.

The formula to calculate the unit vector of the symptom vector u is as follows:

$$\frac{\left\{\left\{u\right\}-\bar{\bar{u}}\right\}}{\left|u\right|}.$$

Here, $\left\{\left\{u\right\}-\bar{\bar{u}}\right\}$ is the difference vector of symptom vector u. |u| is the size of the symptom vector u (Zhou et al., 2015; Sapna and Renuka, 2017).

To evaluate the unit vector v, we use

$$\left(\frac{\left\{\left\{v\right\}-\bar{\bar{v}}\right\}}{\left|v\right|}\right),$$

$\left\{\left\{v\right\}-\bar{\bar{v}}\right\}\;$ is the difference vector of symptom vector v (Zhou et al., 2015; Sapna and Renuka, 2017). |v|  is the size of the symptom vector v (Zhou et al., 2015; Sapna and Renuka, 2017).

(4) Calculating correlation (similarity) of the two unit vectors.

The dot product of the unit vectors is calculated by using (Zhou et al., 2015; Sapna and Renuka, 2017)

$$\left(\frac{\left\{\left\{u\right\}-\bar{\bar{u}}\right\}}{\left|u\right|}•\frac{\left\{\left\{v\right\}-\bar{\bar{v}}\right\}}{\left|v\right|}\right),$$

$\left\{\left\{u\right\}-\bar{\bar{u}}\right\}$ is the difference vector of feature vector u. |u| is the length of the feature vector u (Sapna and Renuka, 2017; Zhou et al., 2015). $\left\{\left\{v\right\}-\bar{\bar{v}}\right\}$ is the difference vector of feature vector v (Zhou et al., 2015). |v| is the length of the feature vector v (Adams et al., 2018; Zhou et al., 2015). If the calculated coefficient of correlation is equal to 1, then the two images are absolutely identical (Kaur et al., 2012; Sapna and Renuka, 2017; Adams et al., 2018). If the calculated coefficient of correlation is equal to 0, then the two images are completely uncorrelated (Kaur et al., 2012). If the coefficient of correlation is calculated as value equal is –1, then the two images are completely anti-correlated (Kaur et al., 2012; Sapna and Renuka, 2017).

## Experimental Results and Comparisons

The images of the different types of infected leaf images of the G-4 (Guntur-4) variety before and after segmentation of powdery mildew (whitefly) and *Fusarium* wilt (yellow) are displayed below (Wang & Chu, 2009; Jung et al., 2017).

**FIGURE 3:**
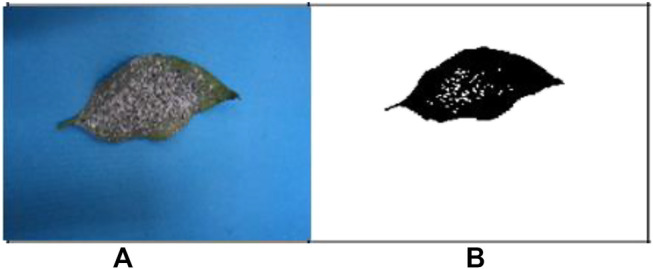
**(A)** Original G-4 chili powdery mildew (whitefly) leaf and **(B)** FCM-segmented chili powdery mildew (whitefly) leaf. Example images acquired after segmentation of **(A,B)**.

**FIGURE 4:**
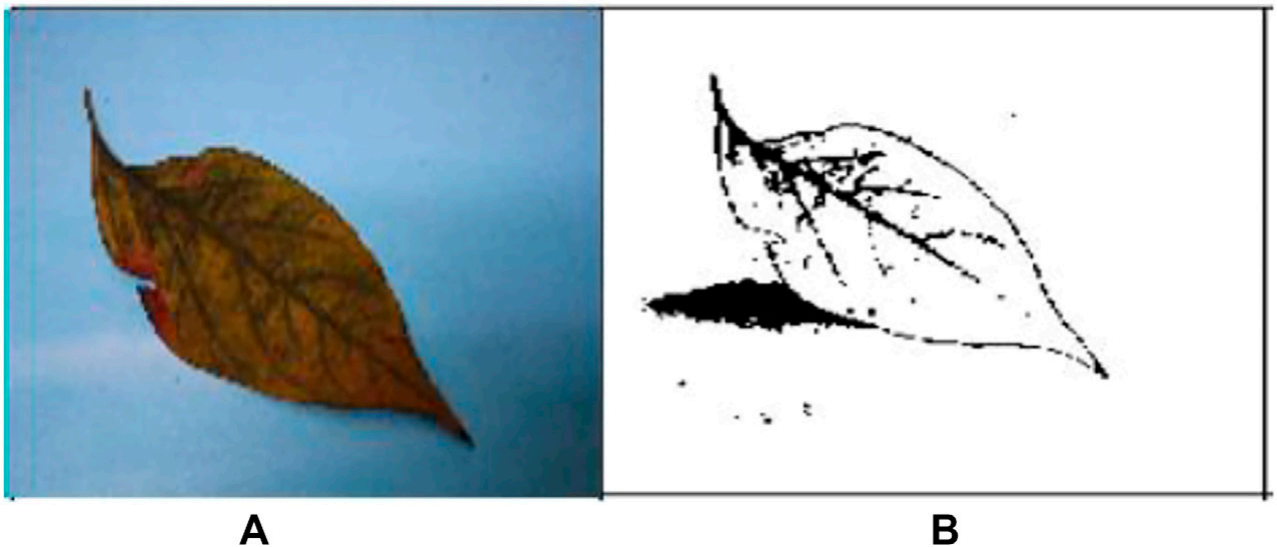
**(A)** Original G-4 chili *Fusarium* wilt (yellow) leaf and **(B)** FCM-segmented chili *Fusarium* wilt (yellow) leaf.

Figures 3A,B are the images from before and after segmentation of powdery mildew (whitefly), and Figures 4A,B are the images from before and after segmentation of *Fusarium* wilt (yellow).

Table 1 shows the distribution of the total data set acquired, of the diseased G-4 chilli plant images.

**TABLE 1:** Acquisition and distribution of the total dataset of disease in G-4 chili plants.

Bacterial leaf spot	Powdery mildew (whitefly)	Chili leaf curl	*Fusarium* wilt (yellow)	Healthy leaf
1,554	1,568	1,570	1,577	1,581

Table 2 shows the feature vector of each type of leaf image extracted based on moments.

**TABLE 2:** Moment—symptoms/features extracted of G-4 leaf images.

Bacterial leaf spot	Powdery mildew (whitefly)	Chili leaf curl	*Fusarium* wilt (yellow)	Healthy leaf
–47.378,046	–44.862,024	–3.708,196	37.780,514	359.94998
–47.235,391	–29.878,586	36.473,097	–86.192,284	87.989,614
–47.378,046	–44.862,024	–3.708,196	37.780,514	359.94998
14.092269	–9.678,214	70.223,784	49.523,289	–6.8,780,067
9.316,937	–9.736,148	19.811,641	1.270,926	–44.44913
9.092269	–9.678,214	70.223,784	49.523,289	–6.878,006
–4.650,409	–3.364,313	9.549,012	9.396,945	68.859,820
–3.637,987	–2.976,504	8.901,379	–8.602,093	25.222,793
–4.650,409	–3.364,313	9.549,012	9.396,945	68.859,820
–20.266,736	22.720,311	–85.743,287	207.250,731	–428.03072
–20.186,063	9.525,725	–28.143,753	–0.925,072	–194.2108
–20.266,736	22.720,311	–85.743,287	207.250,731,126.487,537	–428.0307
6.248,258	6.626,609	38.797,842	33.948,290	451.11662
7.183,165	5.369,558	16.421,145	126.487,537	150.00430
7.248,258	7.626,609	38.797,842	22.855,400	451.11662
–1.183,399	1.511,968	–6.394,242	1.144,891	–106.6528
–2.181,655	1.492,900	–2.245,134	22.855,400	–34.47643
–1.183,399	1.511,968	–5.394,242	17.039310	–106.65282
–7.811,914	–4.071717	–26.751,533	–15.674,799	227.87910
–7.834,569	–0.205,330	–3.841,514	17.039310	33.943,961
–7.811,914	–3.071717	–26.751,533	10.350,127	227.87910
2.013364	–0.479,268	10.682,656	–0.513,281	–34.464,554
2.029504	–0.190,962	3.409,589	10.350,127	–13.414,349
2.013364	–0.479,268	10.682,656	1.869,517	–34.464,554
–0.424,560	–1.115,643	–1.940,388	–0.910,716	45.598,194
–0.425,489	–0.053978	–0.025937	1.869,517	8.1,003,524
–0.424,560	–0.115,643	–1.940,388		45.598,194

Tables 3, 4 show the performance analysis using evaluation metrics (Sufola et al., 2016c). The result of testing shows that the proposed technique has an accuracy of 97.56%.

**TABLE 3:** Performance analysis using evaluation metrics.

Technique	Sensitivity (%)	Specificity (%)	Accuracy (%)
SVM technique	76	72	74
Proposed technique on moments	98.34	95.98	97.56

**TABLE 4:** Performance analysis using evaluation metrics.

Technique	True positive	False negative	False positive	True negative
SVM technique	1083	342	399	1026
Proposed technique	1897	32	37	884

**FIGURE 5:**
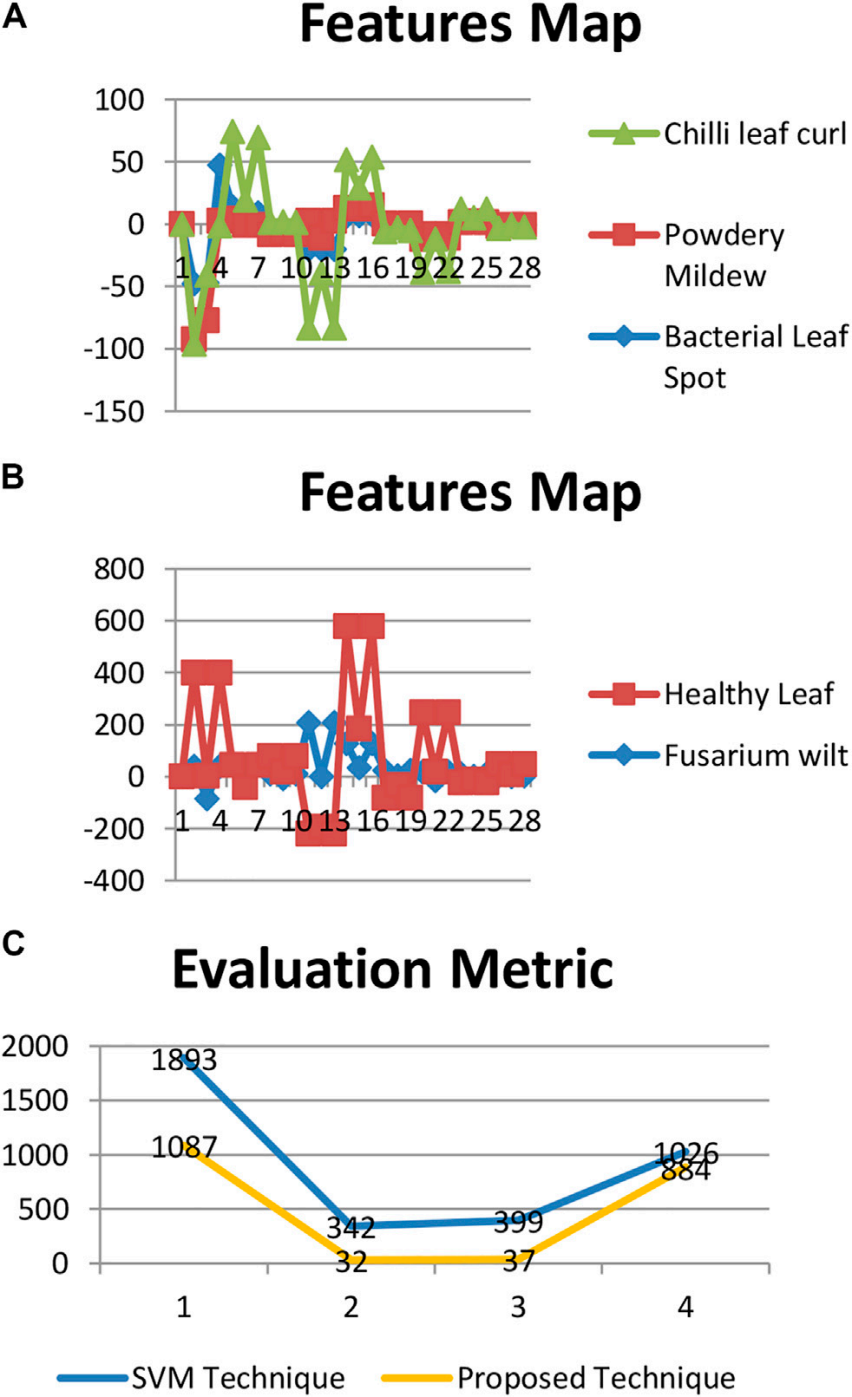
Evaluation metrics of the techniques. **(A)**: Feature map of bacterial leaf spot, powdery mildew (whitefly), and chili leaf curl. **(B)**: Feature map of *Fusarium* wilt (yellow) and healthy leaf.

Figure 5 shows the evaluation matrices of the support vector machine technique and the moments technique used.

**FIGURE 6:**
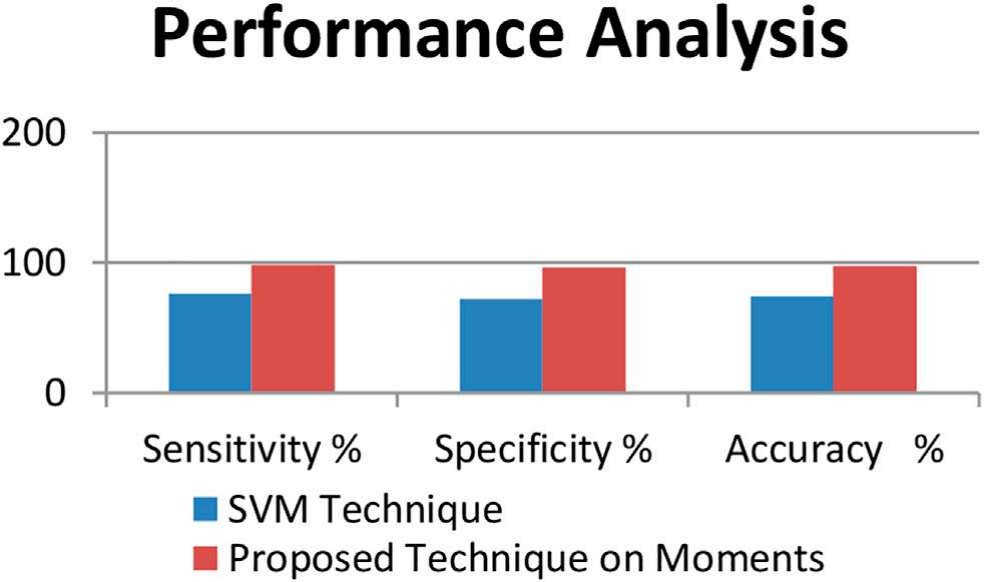
Performance analysis of the techniques.

Figure 6 the representation of the performance analysis of both the techniques used.

## Conclusion

Identification and detection of disease in G-4 (Guntur-4) variety chili leaves are useful for the farmers for detecting the diseases (Sufola et al., 2016d). The proposed system will reduce the errors which can occur during manual detection of diseases (Barolli et al., 2020).

This proposed system would be useful for farmers and scientists for fast detection of diseases. The above study shows that the proposed technique identifies the G-4 chili leaf diseases with an accuracy of 97.56%.

## Data Availability

The original contributions presented in the study are included in the article/Supplementary Material. Any further inquiries can be directed to the corresponding author.
